# Efficient and automated large-scale detection of structural relationships in proteins with a flexible aligner

**DOI:** 10.1186/s12859-015-0866-8

**Published:** 2016-01-05

**Authors:** Fernando I. Gutiérrez, Felipe Rodriguez-Valenzuela, Ignacio L. Ibarra, Damien P. Devos, Francisco Melo

**Affiliations:** Departamento de Genética Molecular y Microbiología, Facultad de Ciencias Biológicas, Pontificia Universidad Católica de Chile, Alameda 340, Santiago, Chile; Centre for Organismal Studies (COS), Heidelberg University, Heidelberg, Germany; Centro Andaluz de Biología del Desarrollo (CABD), Universidad Pablo de Olavide, Sevilla, Spain

**Keywords:** Protein structure comparison, Protein structure search, Flexible structural alignment

## Abstract

**Background:**

The total number of known three-dimensional protein structures is rapidly increasing. Consequently, the need for fast structural search against complete databases without a significant loss of accuracy is increasingly demanding. Recently, TopSearch, an ultra-fast method for finding rigid structural relationships between a query structure and the complete Protein Data Bank (PDB), at the multi-chain level, has been released. However, comparable accurate flexible structural aligners to perform efficient whole database searches of multi-domain proteins are not yet available. The availability of such a tool is critical for a sustainable boosting of biological discovery.

**Results:**

Here we report on the development of a new method for the fast and flexible comparison of protein structure chains. The method relies on the calculation of 2D matrices containing a description of the three-dimensional arrangement of secondary structure elements (angles and distances). The comparison involves the matching of an ensemble of substructures through a nested-two-steps dynamic programming algorithm. The unique features of this new approach are the integration and trade-off balancing of the following: 1) speed, 2) accuracy and 3) global and semiglobal flexible structure alignment by integration of local substructure matching. The comparison, and matching with competitive accuracy, of one medium sized (250-aa) query structure against the complete PDB database (216,322 protein chains) takes about 8 min using an average desktop computer. The method is at least 2–3 orders of magnitude faster than other tested tools with similar accuracy. We validate the performance of the method for fold and superfamily assignment in a large benchmark set of protein structures. We finally provide a series of examples to illustrate the usefulness of this method and its application in biological discovery.

**Conclusions:**

The method is able to detect partial structure matching, rigid body shifts, conformational changes and tolerates substantial structural variation arising from insertions, deletions and sequence divergence, as well as structural convergence of unrelated proteins.

**Electronic supplementary material:**

The online version of this article (doi:10.1186/s12859-015-0866-8) contains supplementary material, which is available to authorized users.

## Background

Structural comparison between proteins is a fundamental and common practice in structural biology with many applications, such as the identification of new domains, the classification into structural families and the detection of evolutionary relationships between protein structures that cannot be found by sequence comparisons. For example, the homology between prokaryotic and eukaryotic cytoskeletal filaments (FtsZ/Tubulin and MreB/Actin) or the paralogy between proteins such as hemoglobin and myoglobin where only revealed once the 3D structures of these proteins were solved and compared [1, 2]. Since the determination of the first structures in the 1970s to the present day, the number of solved protein structures in the Protein Data Bank (PDB) has continued to grow at an exponential rate, with more than one hundred thousand structures available today. To facilitate the organization and analysis of this large amount of information, different structure comparison methods and tools have been developed [3]. However, the rise in number of known structures makes the comparison of query structures against the database increasingly costly (both for time and computational requirements) using existing tools.

Depending on the representation of proteins, current structural alignment methods use two main approaches: methods based at the level of residues or Cα atoms (DALI, Structal, TopMatch, MAMMOTH, CE, MUSTANG, FATCAT, TM-align) [4–11] or based on secondary structure representations (VAST, SSAP, GANGSTA+, QP tableau search) [12–15]. One of the major advantages of methods based on secondary structure representations is that they are generally faster, as there is typically at least one order of magnitude fewer secondary structure elements than residues within a protein. However, residue-based methods are generally more accurate [16].

Structure comparison methods are increasingly successful at detecting more divergent relationships [3]. Significant improvements have also been achieved in terms of speed when searching against large databases [17]. Despite this success, current structural comparison tools have a few major drawbacks that limit their utility for detecting cases of remote homology where protein structures might have diverged considerably. First, they treat proteins as rigid bodies and cannot accommodate the large structural variations observed over long evolutionary divergence, for example, the relationship between the nucleoporins and vesicle coats [18]. Additional structural variations that might be due to protein flexibility or allosteric transitions are difficult to detect with the current methods. Finally, they are usually restricted to the comparison of individual domains and do not consider multi-domain proteins. How many distant structural relationships remain undetected because the tools are not sensitive enough? Our goal was to detect protein structure similarities that are beyond the reach of current tools based on rigid body superposition and, at the same time, to be able to do it efficiently and with competitive accuracy.

To that end, we have developed an efficient flexible aligner tool to compare protein structures based on matrices that contain a simple description of the geometrical arrangement of secondary structure elements. Arthur Lesk was the first to describe a tabular representation, which comprises the information about the relative orientation of the elements of secondary structure (interaxial angle) using a coarse-grained and discrete double quadrant codification [19]. The concept is that the sequential order of secondary structure elements and the geometry of interacting pairs capture the essence of the protein fold. The secondary structure elements and their respective angles and distances can be encoded in a matrix. The secondary structure elements are recorded in order of appearance along the main diagonal of the matrix. Each off-diagonal position contains the angles and distances between the pairs of secondary structure elements. The comparison of these matrices allows a faster structural matching than when using a protein representation at the residue/atomic level. However, secondary structure geometry matrices comparison is an NP-hard problem. Various implementations to solve this problem have been presented, including quadratic and linear integer programming [15, 20, 21]. Those methods are very precise at extracting maximally similar sub-matrices, but this is at the expense of speed when comparing against a large number of matrices such as the complete PDB database. In 2008, Konagurthu proposed the TableauSearch method to detect similarities between matrices using two steps of dynamic programming [20]. TableauSearch is faster than previous methods, but this comes at the expense of accuracy and of lacking the ability to find local matches as compared to global ones [15]. This method is not limited to element pairs that are in contact and uses the scheme previously proposed by Lesk described above [19].

We present and release here a new computer application called MOMA (from MOrphing & MAtching). This tool relies on a new algorithm that incorporates several innovations, which are: 1) it considers the continuous value of the angles instead of the discrete and coarse-grained quadrant codification proposed by Lesk and implemented in TableauSearch; 2) the incorporation of a user-defined maximum distance cutoff to consider contacts between secondary structure elements, 3) a modified two-step dynamic programming algorithm that allows for the maximization of the rigid union of several local and compatible structural matches and 4) a new procedure to solve the integration of several rigid and globally incompatible local matches into a flexible and global solution. This new algorithm, as implemented in MOMA computer application, results in a fully automated and highly efficient global flexible structural aligner, which is able to find structural similarity between distantly related proteins with high accuracy.

## Results and discussion

### Overview of the new method

**Fig. 1:**
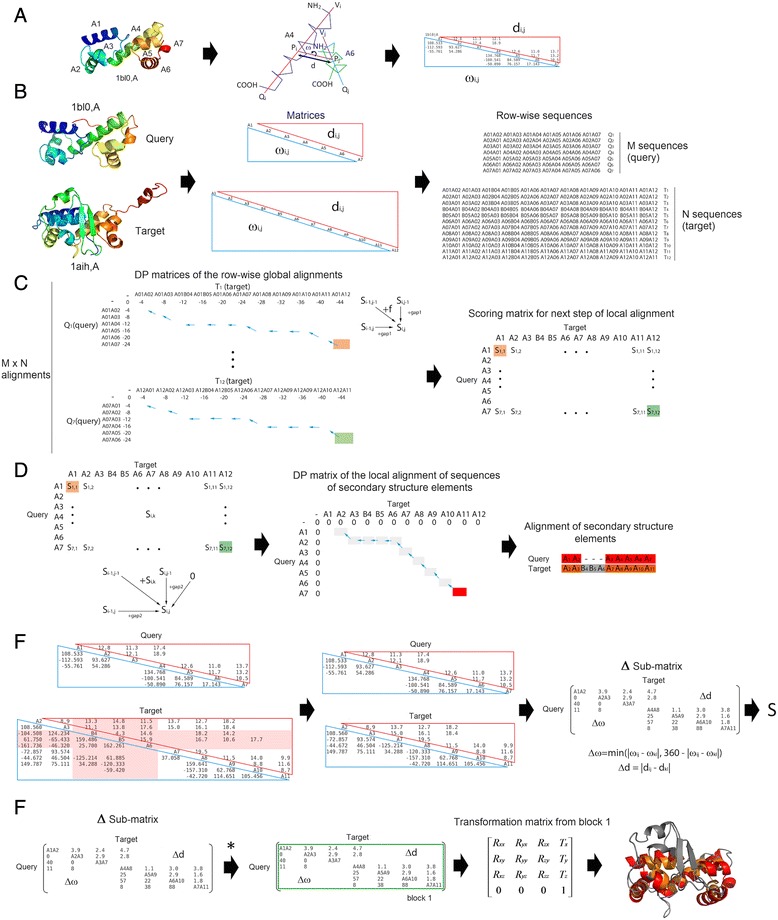
Flowchart of the method as implemented in MOMA. **a** Example of structure of MarA (PDB code 1bl0) and the matrix representation of its folding pattern. The relative orientation of any two secondary structural elements (for example, A4 and A6 helices) is specified by the angle (w) between the vectors along their axes (*left bottom of the matrix*). This is recorded only for those SSE pairs found in close proximity (d < D), as measured by the distance (d) between midpoints of the vectors (*upper right of the matrix*). **b** These matrices are built for the query (1BL0 chain A) and the target (1AIH chain A) structures. After that, row-wise matrices containing all possible SSE pairs in each structure are also built. Query and target proteins render matrices of [M, M-1] and [N, N-1] pairs, where M and N correspond to the total number of SSEs found in the query and target structures, respectively. **c** A first step of global or semi-global dynamic programming (DP) algorithm is executed to build DP matrices for each query row against each target row, thus generating a total of *MxN* DP matrices. In this step, scoring rules and restraints based on angular and distance information of all SSE pairs in each structure are used (see Methods for details). From each DP matrix, only the maximum score value is selected and recorded into a new scoring matrix that is going to be used in a second and final step of a dynamic programming algorithm. In the case of a global alignment, this value is obtained from the bottom right cell of the DP matrix. In the case of the semi-global alignment, this value is obtained from the most right column or the most bottom row of the DP matrix. **d** A local dynamic programming algorithm and the previously built scoring matrix are now used to align the secondary structure elements of the query and the target structures. **e** Unaligned SSE elements from the query and target structures are removed from the initial 2D matrices, thus rendering two matrices of identical dimensions, which can now be compared directly. A delta sub-matrix is built and from it a global matching score calculated (see Methods for details). **f** Finally, a new algorithm (*) is used to infer the list of all incompatible rigid local matches (*blocks*), which are independently superposed with the Kabsch algorithm. In this particular and simple example only one local match or block is found. Details of the algorithm for finding local matching blocks are provided as Supplemental Material (Additional file 1: Figure S1). The resulting superposition is represented with aligned elements in red (*query*) and orange (*target*). Residues not aligned are displayed in grey color

This article describes a fully automated and highly efficient method for the flexible comparison of two protein structure chains. The method relies on the matching of secondary structure elements between the protein chains, based on a two-step dynamic programming algorithm that combines local and global matching procedures. The results obtained when applying this method consist on a single and global structural alignment that integrates all rigid local matches found between the two input protein structures. A general overview of the method is provided in Fig. 1. A detailed description of each step of the method is provided in Methods section.

### Calibration of parameter values

The results of our method, as implemented in MOMA, strongly depend on the value of three parameters, which are the constant that limits the score calculated from the angular difference (C) and the gap-opening penalties for the two steps of dynamic programming (g1 and g2). By optimization of the different combinations of these parameters, we found that the best results were obtained with a C constant value of 45 and a gap-opening penalty of −4 for both steps of dynamic programming (Additional file 1: Table S1). With these parameter values, only 2 out of 100 alignments from HOMSTRAD database have a QS index smaller or equal to 0.5 and the average QS index was 0.9436 (Additional file 1: Table S2). The failure of MOMA to correctly align the corresponding SSE pairs in these two cases is due to an inaccurate assignment of secondary structure elements by DSSP computer program. In some cases, DSSP does not assign the exact start and end points of SSEs. In other cases, long helices and strands with some bending are split into two or more non-contiguous SSEs [21].

Another relevant parameter in the matrix comparison step of our method is the distance cutoff (D) used to define SSE pairs in contact [15]. We tested different values of distance thresholds in the HOMSTRAD set to define the best performing one (Additional file 1: Figure S1 and Table S1). If the distance cutoff value was smaller or equal than 12 Å, several matrices could not be aligned because too few SSE pairs were considered (ie. few contacts are found near the main diagonal of the matrix). Most of the information required to identify a folding pattern is contained in adjacent positions near the main diagonal in the matrices [22].

On the other hand, if the distance cutoff was set to values greater than 20 Å, the average QS index decreased (Additional file 1: Table S1). Therefore, a value of 20 Å was finally used as the maximum distance cutoff to define a contact between two SSEs.

After fixing the previous parameter values, and to evaluate if the raw score reported by MOMA was better than the relative similarity score, we then carried out searches using the seven most common folds as a query against a subset of 19,602 domains from ASTRAL 2.03 (95% sequence-identity cutoff; for details see Methods). The ROC curve analysis of these two scores showed that the relative similarity is slightly better than the raw score (Additional file 1: Figure S2 and Table S3). Thus, we defined relative similarity as the measure to be used for fold assignment by default in our method, as implemented in MOMA.

### Testing the new method

As a first test of our method with the fixed parameter values described above, we used as a query the seven most common folds and searched against the 19,602 domains in ASTRAL 95 % sequence identity dataset. ROC analysis of structure similarity matching results shows that, irrespectively of the query, the method has an excellent performance in terms of accuracy at the fold, family and superfamily levels (Additional file 1: Table S3). Execution time increases exponentially with the total number of SSE elements assigned in the structures (Additional file 1: Figure S4).

### Benchmarking with other methods

The representative set of 100 protein queries was compared against the ASTRAL 2.03 40 % sequence identity dataset (which contains a total of 11,121 domains; for details see Methods) with SHEBA, YAKUSA, QP tableau search, GANGSTA+, Structal, TopMatch and MOMA computer programs. The performance of these methods was assessed by ROC curve analysis based on the normalized scores reported by each of them and adopting the SCOP classification as the gold standard [23]. We also measured the execution time required by these computer programs to perform a search against the full ASTRAL dataset of 11,121 domains with the 100 query structures.

In terms of AUC and maximum accuracy values, both at the fold and superfamily levels, Structal, TopMatch and MOMA are the best performing methods, followed by GANGSTA+, QP tableau search, SHEBA and Yakusa (Table 1; Additional file 1: Figure S3). In terms of accuracy, at the fold and superfamily levels, MOMA has the best performance among methods that use a geometric secondary structure representation of 3D protein structure such as QP tableau search and GANGSTA+, or when compared to currently the fastest methods for 3D structure matching such as YAKUSA and SHEBA. MOMA requires a variable amount of time to complete the search, which depends on the number of SSEs present in the matrix (Additional file 1: Figure S4), but in this large benchmark set MOMA is much faster than all tested methods (at least by one or two-three orders of magnitude faster than most of the tested methods) (Table 1).

**Table 1:** Performance benchmark analysis of MOMA with different methods

Methods	AUC		ACC		^*^fp		^*^tp		time
	Fold	Superfamily	Fold	Superfamily	Fold	Superfamily	Fold	Superfamily	
Structal	0.956	0.969	0.902	0.919	0.076	0.060	0.880	0.898	10d 21h (1,842x)
TopMatch	0.955	0.974	0.883	0.911	0.121	0.069	0.887	0.891	2d (339x)
MOMA	0.940	0.956	0.872	0.889	0.139	0.113	0.884	0.891	8m 28s (1x)
GANGSTA+	0.916	0.911	0.845	0.851	0.101	0.058	0.791	0.761	5d 6h 49m (895x)
QP tableau search	0.877	0.918	0.791	0.831	0.224	0.188	0.805	0.850	2d 7h 27m (391x)
SHEBA	0.870	0.889	0.841	0.875	0.052	0.042	0.734	0.793	6h 51m (48x)
FATCAT flexible	0.837	0.911	0.743	0.825	0.220	0.211	0.706	0.862	27d 2h 38m (4,614x)
YAKUSA	0.790	0.858	0.727	0.794	0.155	0.088	0.609	0.677	48m (5.7x)

Area under ROC curve (AUC), maximal accuracy (ACC), false positive (fp) and true positive (tp) rates for each method are reported (^*^these values are calculated at the same threshold that gives the maximum accuracy reported as ACC). The execution time needed to compare the 100 queries against the 11,121 domains in the ASTRAL SCOP 40% sequence identity dataset is shown in the last column of the table. Execution times are reported in seconds (s), minutes (m), hours (h) and days (d) (in parenthesis, the speed gain factor of MOMA when compared to other methods is displayed, where “x” means number of times faster)

A detailed analysis of ROC curves reveals that SHEBA is a more specific classifier than MOMA, GANGSTA+ and QP tableau search, exhibiting a very low rate of false positives at the fold and superfamily levels. However, these methods have a higher sensitivity when compared to SHEBA. GANGSTA+ has an excellent performance and is better than QP tableau to search for proteins with the same fold, but QP tableau search is better than GANSGTA+ at a rate of false positives >0.6 for the superfamily level.

At the fold level, Yakusa is always worst than SHEBA, QP tableau search, GANGSTA+ and MOMA. However, Yakusa has a slight advantage than SHEBA at a rate of false positives >0.5 for the superfamily level.

The statistical analysis of the AUC curves reveals that the difference observed in the performance of MOMA with other computer programs is statistical significant at the 95 % confidence level (Additional file 1: Table S4).

As for the running time of each method, MOMA is the fastest of the methods tested. For example, it takes only 8 min and 28 s to search the 100 queries against the whole ASTRAL 40 %, while all other methods take more than 45 min, hours or even days of execution time (Table 1). We note that Structal, GANGSTA+, QP tableau search, and SHEBA are infeasible to run queries on very large datasets, such as the PDB database, which was one of the goals that motivated us to develop this method. Although QP tableau search can calculate the exact solution of the comparison of two matrices and GANGSTA+ can generate non-sequential protein structure alignments based in SSEs, MOMA has a better performance and is much faster than these two methods.

### Biological applications

#### Rigid body shift caused by a rearrangement of domains

**Fig. 2:**
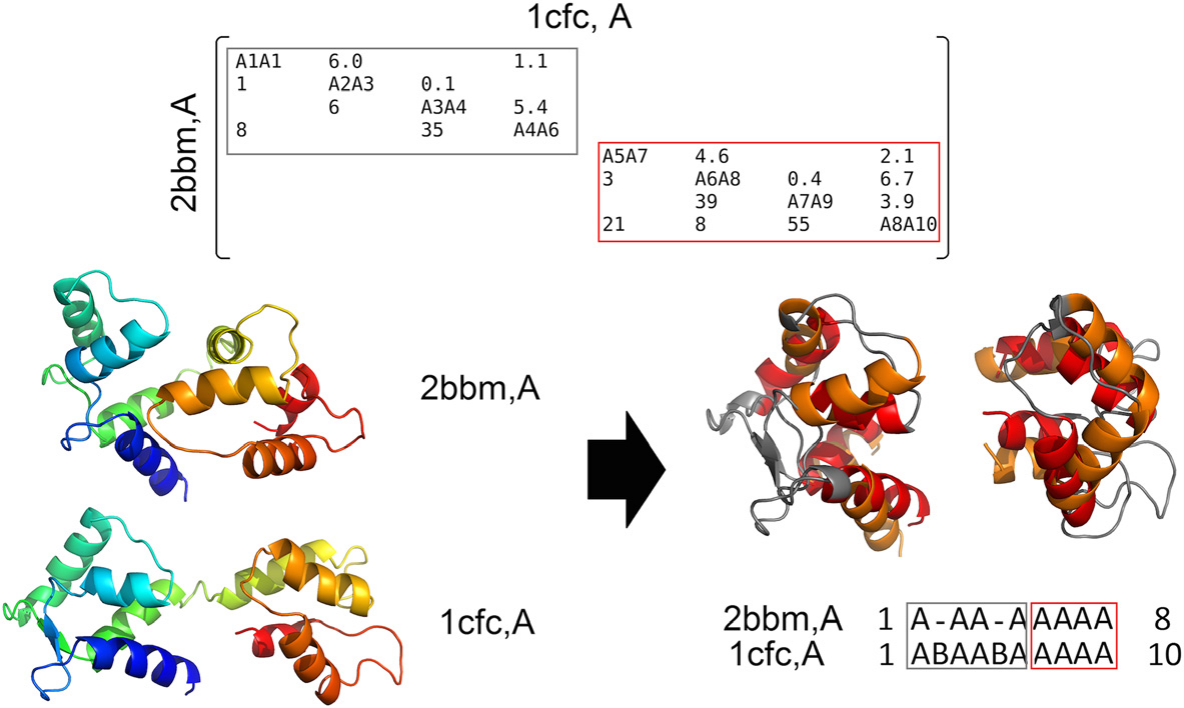
Example of a rigid body shift caused by the rearrangement of two structural domains upon ligand binding. Structure superposition generated with MOMA of the Calmodulin-target peptide complex (the query; PDB code 2BBM, chain A) and the calcium-free Calmodulin (the target; PDB code 1CFC, chain A). Top Panel: The conserved domains are shown in the alignment of SSEs and the respective sub-matrices surrounded by grey and red blocks. Bottom Panel: The two structures and the superposition of their aligned domain pairs are shown respectively in rainbow color representation (*Left*) and with the SSE pairs structurally aligned in red and orange colours (*Right*). Non-aligned residues are shown in grey. The alignment of SSE elements is also represented with aligned blocks highlighted (*Bottom Right*). The structural superposition of these two domain pairs required different rotation matrices and translation vectors. In this example MOMA was executed with the following parameters: g1 = −4, g2 = −4 and C = 90

A well-known case that illustrates an example of rigid body movement between two structural domains is provided by the comparison of structures of calmodulin with and without Ca^2+^ ion (PDB codes and 2bbm and 1cfc, respectively). Both structures have 4 EF-hands, which consist of a helix-loop-helix motif that interact with Ca^2+^and are organized into two distinct globular domains (N-terminal and C-terminal domains) [24]. These two domains are connected by a linker that is unstructured. This specific case is difficult to align due to the flexibility of the 6 loops and of the central linker. In the calmodulin-Ca^2+^structure, the two calcium-binding domains are wrapped around a binding peptide in a “close” conformation while in the Ca^2+^free structure, a rotation around the axis of the linker leaves the two domains in an “open” conformation. Other flexible aligners such as Flexprot [25] and FATCAT [10], required the introduction of four or more rigid-body movements (twists) around pivot points (hinges) to obtain a good superposition of these two structures. In a single step, MOMA is able to automatically detect the conserved N-terminal and C-terminal domains, as shown in the matrix alignment, despite the different relative orientation of the two domains (Fig. 2).

#### Simple but significant structural rearrangement

**Fig. 3:**
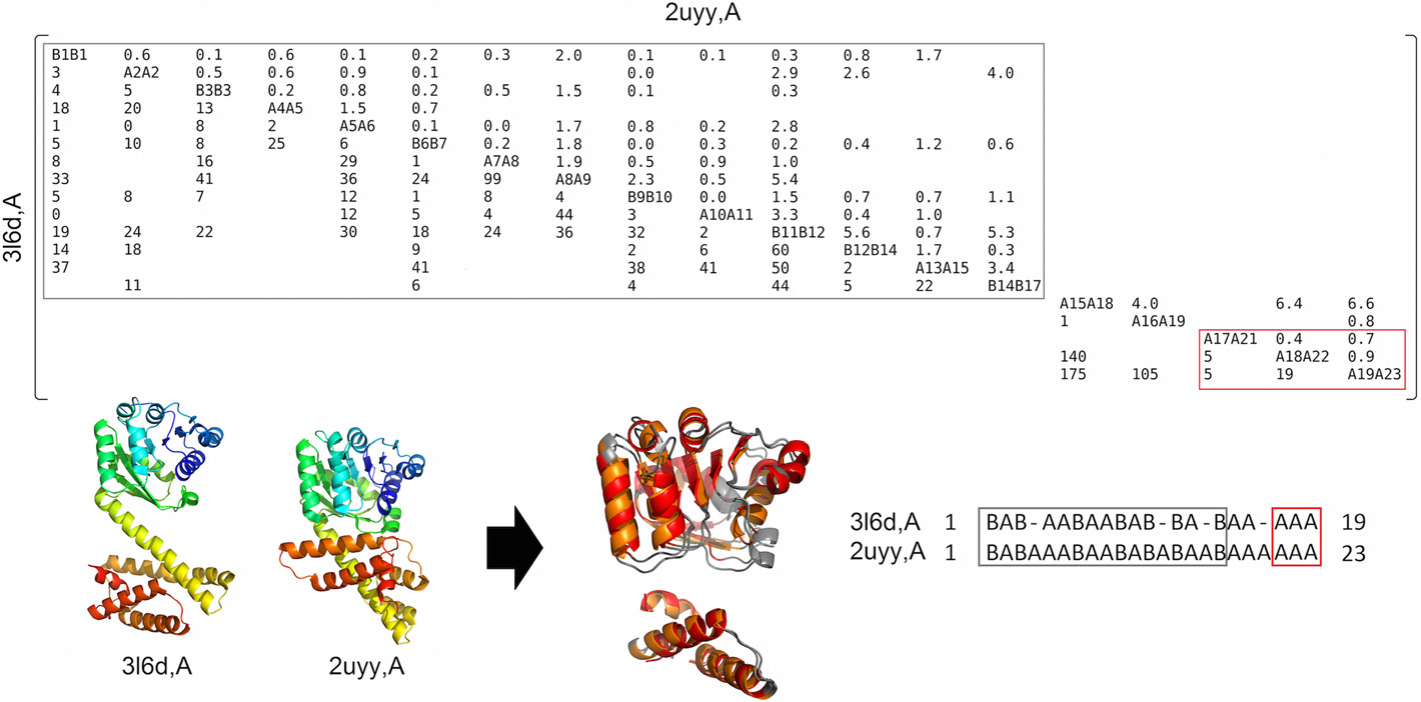
Example of a simple but significant structural re-arrangement. Structure superposition of the putative oxidoreductase from Pseudomonas putida (the query; PDB code 3L6D, chain A) and the human Cytokine-like Nuclear Factor N-Pac (the target; PDB code 2UYY, chain A) generated with MOMA. The conserved domains are shown in the alignment and the sub-matrices remarked by grey and red blocks (*Top Panel*). The two structures are rainbow colored (*Bottom Left*) and the resulting SSE pairs aligned are shown in red and orange colours (*Bottom Middle*). The alignment of SSE elements is also represented with aligned blocks highlighted (*Bottom Right*). Each superposition was carried out with different rotation matrices and translation vectors. In this example MOMA was executed with the following parameters: g1 = −4, g2 = −4 and C = 45

The case of two functionally unrelated proteins illustrates the capacity of MOMA to obtain the global alignment of two structurally similar domains whose relative orientation is not conserved. The putative oxidoreductase from *Pseudomonas putida* (PDB code 3l6d) and the human Cytokine-Like Nuclear Factor N-Pac (PDB code 2uyy) share two almost identical structural domains, which are separated by a connecting linker (Fig. 3). This linker is composed by two or three helices in bacterial and human proteins, respectively. The differential number of helices present in the linkers orients the two domains differently in the bacteria and human proteins. This simple structural rearrangement is a challenging problem for structural similarity detection methods, because the orientation of the two domains is different in both proteins. Rigid structure comparison tools can only identify the matching of these domains as two separate solutions, in the rare cases where more than one solution is reported (ie*.* TopMatch).

The power of MOMA resides in the fact that the structural similarity between both structural domain pairs is automatically detected and reported in a single step. In addition, the source of the conformational difference is also readily detected and highlighted in the alignment matrix (ie*.* helix 20 of 2uyyA cannot be aligned to a missing corresponding helix in 3l6dA).

#### Complex structural rearrangement

**Fig. 4:**
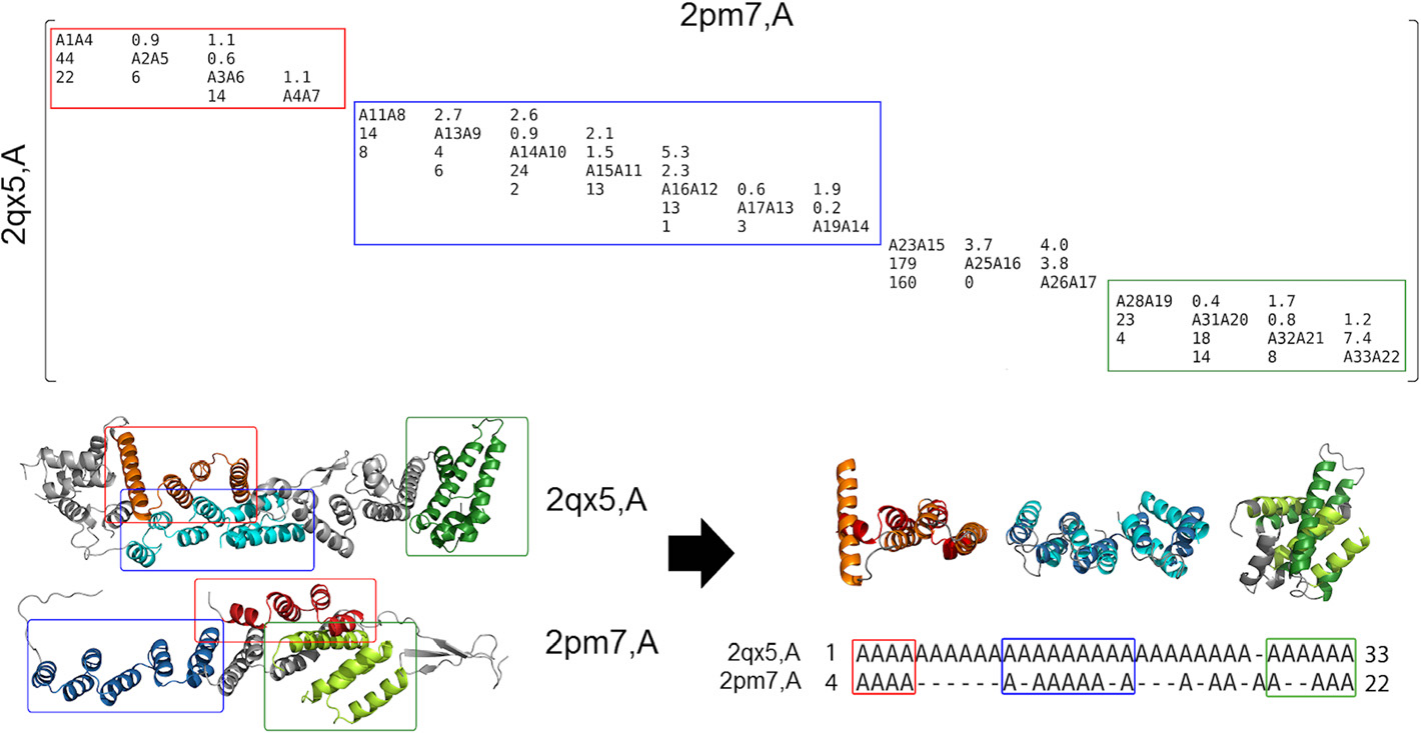
Example of a complex structural re-arrangement. Structural superposition with MOMA of proteins Sec31 of the COPII complex of coat vesicle (the query; PDB code 2QX5, chain A) and the nucleoporin Nic96 (the target; PDB code 2PM7, chain A). Top Panel: The structure matching domains are shown in the alignment of SSEs and the respective sub-matrices surrounded by red, blue and green colored boxes. Bottom Panel: The three independent protein structure sectors that match between the two proteins, identified by MOMA, are highlighted by green, orange and blue colored boxes (*Bottom Left*). The alignment of SSE pairs and the independent structural superposition of the three matching block pairs are shown in red-orange, cyan-blue and light–dark green colours (*Bottom Right*). In this example MOMA was executed with the following parameters: g1 = −5, g2 = −5 and C = 45

A more impressive example of structural rearrangement detection occurs in the case of Sec31 subunit from the COPII coated vesicle complex and the nucleoporin Nic96. Despite a lack of detectable sequence similarity [26], it is now generally accepted that coated vesicles proteins and nucleoporins have a common origin [18, 26]. However, considerable divergence has occurred since the event of gene duplication, up to a point that sequence similarity cannot be detected any longer, even by the most recent and powerful methods [27]. This sequence divergence has had important consequences on the structural conformation, interactions and cages formed in these two proteins [27]. This is the type of structural divergence that we aimed to detect efficiently and automatically, and thus the main motivation behind the development of the new method reported here. Nic96 (PDB code 2qx5) and Sec31 (PDB code 2pm7) are mainly composed of pairs of α-helices that are stacked on each other, hence termed SPAH domain (for Stacked Pairs of Alpha-Helices; also referred to as α-solenoid domain). Both proteins adopt a roughly linear shape that can be divided into three sections of conserved local structure (Fig. 4). However, those three conserved sections are preceded, followed and separated by other sections that exhibit considerable structural deviation. Sections 1 and 2 are separated by a compact globular U-turn in Nic96, while this linker is unstructured in Sec31. The linker between sections 2 and 3 is composed by 9 α-helices in Nic96, but only by 3 α-helices in Sec31. These substantial structural modifications imply that section 1 is interacting only with section 2 in Nic96, while in Sec31 section 1 interacts almost exclusively with section 3. The relative orientation between the three blocks is also very different in both proteins. Despite these considerable global structural differences, the local structural similarity of the three blocks is clear and represents a legacy of their common ancestry [26]. To the best of our knowledge, MOMA is the only existing tool that is able to readily detect this intricate structural conservation in an automated fashion, which was the initial motivation of this work. The result obtained for this example case with MOMA clearly illustrates the power and potential for biological discovery of the new method reported here.

### Strengths and weaknesses of the method

The speed, accuracy and flexible alignment capability of the method described here are their distinctive strengths. The method, as implemented in MOMA computer tool, is able to detect distant structural relationships in proteins in an automated fashion and efficiently, which makes it suitable to search the complete PDB for biological discovery. Among the weaknesses is the fact that MOMA is a single chain and topology-dependent protein structure alignment tool (ie. it depends on the connectivity order of SSEs). Few other tools, such as TopMatch and Structal have the capability of aligning protein structures in a topology-independent manner, but this comes at the cost of a longer execution time (these computer programs are 2–3 orders of magnitude slower than MOMA). TopMatch is the only tool currently available that is capable of aligning multiple protein chains, but the alignments are rigid and not flexible, which is a drawback in order to find domain movements or significant structural re-arrangements as exemplified here.

Structal was the most accurate tool in our benchmark (Table 1; Additional file 1: Table S4). A detailed analysis of the benchmark differences observed between Structal and MOMA shows that out of the 4,340 and 3,882 positive cases reported by Structal and MOMA, respectively, a total of 3,618 positive cases are common to both methods. There are 722 and 264 positive cases reported only by Structal and MOMA, respectively. Out of the 722 positive cases that Structal reports and MOMA fails to detect, 36.1 % is because of topological re-arrangements and 16.7 % is because there are too short or very few SSEs in the structures. In 11.1% of the cases, MOMA fails to detect the positive cases because of large differences in secondary structure definitions between the target and the query structures. It is noteworthy to mention that the use of STRIDE [28] or DSSP to assign SSEs produced, in a general basis, no significant difference on the performance of MOMA in our benchmark test (Additional file 1: Table S5). However, the accuracy of our method does depend directly on the assignment of SSEs, as well as on its use to represent protein structures and on its intrinsic topology-dependency. On the other hand, this simplified representation translates into a significant gain of speed without an important loss in accuracy (MOMA was 1,842 times faster than Structal in our benchmark test, but only 3 % less accurate). Finally, it is important to mention that the method described here produces structural alignments of secondary structure elements and not structural alignments at the residue-level. Therefore, if required, MOMA could be used in a first stage for fast database search on the task of fold or superfamily assignment and then, afterwards and only for positive matches, a more sophisticated software tool able to incorporate topology re-arrangements and to provide residue-level structure alignment, could be executed in a nested and sequential manner.

It is noteworthy to mention that this new method is not only restricted to protein structure comparison and could be implemented for many other applications that require the maximization of global shape matching between two three-dimensional objects with significant conformational variation, provided that those objects can be represented with vectors of different types which are relevant to describe the shape of the object, but with the limitation that vector order is a constraint of the method (ie. the method is topology-dependent).

## Conclusions

We have developed a new structural comparison algorithm based on the spatial arrangement of secondary structure elements and shown that it allows the efficient retrieval of similar folding patterns in database searches. MOMA exhibits a high sensitivity to detect distant structural similarities without compromising its performance at identifying proteins that share a common fold.

In this regard, the development of a new combined global/semi-global and local structural alignment method that relies on a two-level nested dynamic programming algorithm and involves a new scoring scheme based on the continuous angular difference of SSE pairs close in 3D space instead of the previously used discrete quadrant codification, significantly improved the accuracy to find global similarities based on local matches in protein structures.

## Methods

### Protein structure and benchmark datasets

We used different protein structure datasets to first optimize the value of some parameters and then to evaluate the implementation of our method. First, to calibrate internal parameter values of the program, we used a subset of 100 pairwise structural alignments obtained from HOMSTRAD database [29] as previously described [30]. We kept only those alignments with a percentage sequence identity equal or less than 25 % and an average sequence length equal or greater than 150 residues (Additional file 1: Table S6). In this calibration process, a measure of similarity (the QS index) was maximized (see below). Second, to define the similarity score used and reported by our method, we used a small set of seven protein structures that represent the most common folds according to TOPS database [15, 20]. These seven proteins were used as a query to search against the ASTRAL SCOPe 2.03 95 % sequence identity protein domain database that contains 19,602 entries [31] (released October 2013). Receiver operating characteristic (ROC) curve analysis was performed and the area under the curve (AUC) measure was used to define the best performing score for classifying at the fold, family and superfamily level the query structures (see below).

Finally, to evaluate the performance of MOMA and other methods at classifying protein structures at the fold and superfamily levels, we used a representative set of 100 proteins extracted from the ASTRAL SCOPe 2.03 95 % sequence identity protein domain database described above (19,602 entries). These 100 proteins were used as a query to search for common structural matches against a non-redundant subset obtained from ASTRAL SCOPe 2.03 protein domain database [31] (released October 2013) with a 40 % sequence identity cutoff, which contains a total of 11,121 entries, none of them being any of the 100 query proteins. In this benchmark, we also carried out ROC curve analysis to assess and compare the performance of the methods (see below). All datasets described in this paper are available as supplementary data at: http://melolab.org/supdat/moma.

### Computer software and methods

We used the DSSP program [32] to assign the secondary structure of proteins and the Numpy Python library to calculate the vectors and interaxial angles between the secondary structure elements. Moreover, we evaluated and compared MOMA against six methods based on their performance at classifying protein structures with similar folds or belonging to the same superfamily. The tested software implementing different methods were TopMatch [6], SHEBA [33], Yakusa [34], QP tableau search [15], Structal [5, 35], FATCAT [10] and GANGSTA+ [14]. These computer programs were used with their default parameter values. All calculations were carried out using an Intel Core i7 2.64 GHz processor with 12 GB RAM memory and Ubuntu 13.04 Linux operating system.

### Method description

To construct a 2D matrix from the 3D structure of a protein, the secondary structural elements (SSE) are assigned with the DSSP program, version 2.0.4 [32]. Only α-helices and β-strands with more than four and three residues, respectively, are considered in the analysis. Different types of α-helices (π, 3_10_ and α) are treated equivalently and always assigned as a common α-helix type. Next, each secondary structure element is represented as a vector from its amino to carboxyl terminus by linear square fitting of an axis through the Cα coordinates with the singular value decomposition method [36].

After that, the interaxial angle between each pair of SSE vectors and the Euclidean distance between the midpoints of the axes is computed (Fig. 1a). The interaxial angle (ω) is the shortest rotation (clockwise or anticlockwise) required for the reorientation of the nearest vector that eclipses the farther vector, its value is restricted between −180° and 180° and was calculated as previously described [21]. Finally, the angle and distance between each pair of SSEs are recorded in the two halves of a 2D matrix: 1) the angle half-matrix and 2) the distance half-matrix. Two SSEs are only considered to be in contact if the distance between the midpoints of their linear axes is below a user-defined cutoff (see below). The diagonal positions are labeled by the elements of secondary structure, numbered by order of appearance in the amino acid sequence, from NH2 to COOH terminus (where ‘A’ stands for α-helix and ‘B’ for β-strand). All off-diagonal positions in the matrix are either blank, if the SSE pairs are not in contact, or they contain the observed angle or distance value of the corresponding SSE pair (Fig. 1a).

To compare 2D matrices of different size, we implemented a different method than that of TableauSearch [20] for submatrix matching. Our method aligns the two matrices with a nested dynamic programming algorithm. The first step of the method is aimed at discovering putatively equivalent SSE pairs by comparing each row in the query matrix with each row in the target matrix, with a global or semi-global alignment and a constant gap opening penalty value model (denominated g1). The rows are treated as linear sequences of SSE pairs (Fig. 1b). Therefore, each element in a row represents a pair of different SSEs in a protein. If the query and target structures contain *M* and *N* elements of secondary structure, then a total of *M* and *N* rows are generated from the query and target structures, respectively. Consequently, in this step of the method, a total of *MxN* global or semi-global alignments are calculated (Fig. 1c).

Semi-global alignment is similar to global alignment, in the sense that it attempts to align the two sequences entirely. The difference between both methods lies in the way the alignments are scored. Semi-global alignment assigns no cost to opening end gaps in the alignment [37]. This alignment type selection depends on the difference in the number of SSEs identified in the query and target structures (ie*.* the size difference of the matrices). If the maximum ratio of the number of SSEs from the two structures is greater than two, a semi-global alignment is calculated; otherwise, a global alignment is built. We defined a scoring function that takes into account the value of interaxial angle (in degrees) calculated for each pair of SSEs, implicitly incorporating the distance between the two vectors. This function was defined as follows:

1 $$ f\left({\omega}_{\mathrm{i}\mathrm{j}},{\omega}_{\mathrm{k}\mathrm{l}},{\mathrm{d}}_{\mathrm{i}\mathrm{j}},{\mathrm{d}}_{\mathrm{k}\mathrm{l}},{\mathrm{E}}_{\mathrm{i}},{\mathrm{E}}_{\mathrm{j}},{\mathrm{E}}_{\mathrm{k}},{\mathrm{E}}_{\mathrm{l}}\right) = \left\{\begin{array}{l}0,\ {\mathrm{d}}_{ij}>D\ \mathrm{or}\ {\mathrm{d}}_{kl}>D\hfill \\ {}-C,\ {\mathrm{E}}_{\mathrm{i}}{\mathrm{E}}_{\mathrm{j}}\ne {\mathrm{E}}_{\mathrm{k}}{\mathrm{E}}_{\mathrm{l}}\hfill \\ {}-\mathrm{C},\ \varDelta \omega >2\mathrm{C}\hfill \\ {}\mathrm{C}-\varDelta \omega,\ \mathrm{otherwise}\hfill \end{array}\right\} $$

2 $$ \varDelta \omega = \min \left(\left|{\omega}_{ij}-{\omega}_{kl}\right|,360-\left|{\omega}_{ij}-{\omega}_{kl}\right|\right) $$

where E_x_ stands for an element of secondary structure in relative position *x* from NH2 to COOH terminus in the protein chain, which can adopt two possible labels or values: A for alpha helix and B for beta strand; E_i_E_j_ and E_k_E_l_ are SSE pairs in the query and target structure, respectively; ω_ij_ and ω_kl_ are the interaxial angles between the E_i_E_j_ pair in the query structure and between the E_k_E_l_ pair in the target structure, respectively; d_ij_ and d_kl_ are the distances between the E_i_E_j_ pair in the query structure and between the E_k_E_l_ pair in the target structure, respectively; Δω is the minimal angular difference between ω_ij_ and ω_kl_, and C is an angular constant (in degree units). D is the maximum distance allowed to define that two SSEs are in contact (in Angstroms). This function is subjected to several constraints. The first constraint, d_ij_ < D and d_kl_ < D, is introduced in order to avoid false positives when pairs of SSEs in two proteins have a similar interaxial angle, but are found at very different distances in the two structures [15] or found at very large distances in both the query and target structures. It is expected than in these cases there is no direct association between the SSE pairs in the two structures that should be used to infer fold similarity. This restriction is applied if at least one of the pairs is not in contact, as defined by the maximal distance cutoff D (a user-defined parameter). The second constraint, E_i_E_j_ = E_k_E_l_, ensures that two SSE pairs of different types should not be matched (for example, helix-helix with strand-helix or with strand-strand) and the third constraint, Δω < 2C, ensures that the function takes values between C and -C. Finally, the adopted constant gap opening penalty values for the two levels of the dynamic programming algorithm were those resulting from an optimization process using one of the benchmark datasets (see section 2.6 below and Additional file 1).

The optimal score value obtained from each query and target row alignment (Fig. 1c) is taken to generate the scoring matrix that is used in the second alignment step (Fig. 1d), but this time with the local Smith-Waterman dynamic programming algorithm [38]. Here, a different constant gap opening penalty value can be adopted (denominated g2), which is another user-defined parameter required by our method. The alignment of SSE elements between the query and target structures is generated by the usual backtracking procedure (Fig. 1d).

At this point, it is important to mention that this alignment contains the union of all local structurally matching SSEs between the query and target structures, concordant to optimized, but not yet integrated global information of structurally matching SSE pairs. Therefore, the current alignment cannot be directly interpreted as a global structure alignment of two rigid bodies. In the case of highly related proteins this alignment will be accurate, but in the case of proteins with domain movements, rigid body shifts or partial structure matching, the identification of the structural regions to be matched as rigid body shifts by unique geometrical transformations is still needed.

The next step of the method consists on removing all rows and columns corresponding to non-aligned SSEs from both 2D initial matrices, the query and the target, thus rendering two matrices of identical size and shape that can be now compared directly and efficiently, in a one-to-one cell-to-cell manner (Fig. 1e). A unique 2D difference sub-matrix is now produced (called ΔSM or delta sub-matrix), which contains in the diagonal the labels for only those matching SSE pairs between the query and target structure, along with their differences in angle (upper middle triangle) and distance (lower triangle). Only the difference values for SSE pairs below a maximum parameter value, named ΔD, are reported in this difference matrix.

### Structural matching score and similarity measures

A score of overall and integrated structural similarity for the query and target structures is calculated from the 2D difference sub-matrix (Fig. 1e). This score represents an estimation of the global integration of local matches. We calculate a measure of integrated structural similarity based on a Gaussian function that considers the angular difference observed in the matrix. This raw score can be defined as:

3 $$ S = \frac{{\displaystyle \sum_i^N{e}^{-{r}_i^2}}}{\sigma^2},\kern0.5em {r}_i^2=\varDelta {\omega}^2 $$

where r_i_^2^ is the squared angular difference observed between two SSE pairs below distance threshold D, N is the total number of the SSE pairs aligned and σ is the scale parameter that determines the reduction rate of the score as a function of increasing angular difference. If the target structure is structurally equivalent with the query structure (ie. similar matrices), the score is equal to the total number of SSE pairs aligned. With increasing spatial deviation of the angular difference of SSE pairs aligned, the score approaches to 0.

In addition to score S, for comparing proteins of different size, we implemented two normalization functions. One of these functions is the relative similarity, S_r_ [39], which constitutes a global similarity measure between two proteins, and is defined by:

4 $$ {S}_r=100\times \frac{2S}{n_q+{n}_t} $$

where n_q_ and n_t_ are the number of SSE pairs that are in contact in the query and target matrices, respectively, and S is the raw score described above. Another normalization function is the relative cover C_r_ [30] which represents the cover of the structural match in the smallest protein with respect to the largest protein [39], and it was implemented in the following function:

5 $$ {C}_r=\frac{100\times S}{ \min \left({n}_q,{n}_t\right)} $$

The integration of the information from all these score similarity measures allows the detailed assessment of structure similarity between two protein chains, from a local and global perspective, at once.

### Inference of compatible local structural matching

To obtain a flexible and global superposition of two structures, a complete list of rigid local sub-matches between the two structures must be generated (Fig. 1f). Each rigid local sub-match follows a specific geometric transformation (ie. a specific rotation matrix and translation vector pair). To that end, we have implemented an algorithm that infers all local and rigid matches from the 2D difference sub-matrix. The only constraint imposed by this algorithm is that a minimum local match must contain at least three pairs of SSE elements. Briefly, the algorithm follows the diagonal below and adjacent to the main diagonal, checking for the observed Δω values. To initiate a new local matching block, a non-null Δω value equal or smaller than 90° is needed. If the next value is equal or smaller than 90°, the algorithm extends the matching block. If the observed Δω value is absent (ie*.* null), then the block is trimmed. Matching blocks smaller than 3 × 3 are not considered. If the Δω value is larger than 90°, then the adjacent left-row and bottom-column cell values are checked for non-null values equal or smaller than 90°. If this is not fulfilled, the matching block is trimmed. The detailed pseudocode of this algorithm is provided as supplementary material (Additional file 1: Figure S5).

### Integrated visualization of structural matches

Finally, the local matching blocks are superposed in 3D following independent geometrical transformations. To achieve this, the coordinates of the SSE vectors belonging to each local matching block are first extracted. Then, both sets of coordinates are superposed using a particular implementation of the Kabsch algorithm [40], which is based on Lagrange multipliers to solve the optimal superposition problem. This algorithm implementation was proposed by Kearsley and provides an analytical solution based on quaternions to generate the three-dimensional superposition with minimal root mean square deviation [41]. The end result is the flexible global superposition of two structures (Fig. 1f).

### Parameterization of the method

The gap-opening penalties defined in the steps of dynamic programming, C constant and maximum distance cutoff are the most important parameters to compare the SSE matrices. To calibrate these parameters in our method, we aligned 100 homologous protein pairs from HOMSTRAD dataset, carrying out several tests with different combinations of parameter values.

We used the Sorensen-Dice similarity index (QS) [42] to compare the precision of the method to detect equivalent pairs of SSEs in matrix alignments, using as gold standard the HOMSTRAD superpositions. The QS index was defined as:

6 $$ QS=\frac{2\times M}{A+B} $$

where A and B are the number of SSE pairs aligned that were reported by MOMA and HOMSTRAD, respectively, and M is the number of SSE pairs aligned in common. QS index lies between 0 (all SSE pairs aligned by MOMA are different from those reported by HOMSTRAD superposition) and 1 (SSE pairs aligned by MOMA are equal to those reported by HOMSTRAD). In each test, we calculated the average QS index to determine the best combination of parameter values (Additional file 1: Table S2).

### Performance assessment

We performed standard receiver operating characteristic (ROC) curve analysis and adopted the area under the ROC curve (AUC) as the accuracy measure for each method [43]. In these tests, SCOP classification (same fold, superfamily or family) was used as the gold standard to define true positive and true negative instances. Given a protein query and considering the list of hits above a score threshold returned by a search against the datasets, we counted a hit as a true positive (TP) if the structure target had the same SCOP classification level as the protein query. Otherwise, it was classified as a false positive (FP). The statistical significance of the observed differences in classifier performance was calculated with StAR web server (http://melolab.org/star) as previously described [44].

## Additional file

Additional file 1:
**Suplementary data, including:** **Table S1.** Average QS values for best combinations of MOMA parameter values; **Table S2.** Comparison of structural alignments generated by MOMA with those defined in HOMSTRAD; **Table S3.** Benchmark test to assess the performance of MOMA; **Table S4.** Statistical analysis for the benchmark of MOMA with other methods; **Table S5.** Statistical analysis for the benchmark of MOMA with different methods to assign secondary structure; **Table S6.** Set of 100 distant homologous protein pairs obtained from HOMSTRAD database; **Figure S1.** Calibration of distance cutoff using the HOMSTRAD set with the best combination of parameter values; **Figure S2.** ROC curves for the small set of seven most common folds according to TOPS database; **Figure S3.** ROC curves of classification at the SCOP fold and superfamily level; **Figure S4.** Execution time of MOMA; **Figure S5.** Algorithm used for extracting the rigid local matches. (PDF 2793 kb)

## References

[1] Erickson HP (1998). Atomic structures of tubulin and FtsZ. Trends Cell Biol.

[2] van den Ent F, Amos LA, LoÈwe J (2001). Prokaryotic origin of the actin cytoskeleton. Nature.

[3] Hasegawa H, Holm L (2009). Advances and pitfalls of protein structural alignment. Curr Opin Struct Biol.

[4] Holm L, Sander C (1995). Dali: a network tool for protein structure comparison. Trends Biochem Sci.

[5] Gerstein M, Levitt M (1996). Using iterative dynamic programming to obtain accurate pairwise and multiple alignments of protein structures. Proc Int Conf Intell Syst Mol Biol.

[6] Sippl MJ, Wiederstein M (2012). Detection of spatial correlations in protein structures and molecular complexes. Structure (London, England : 1993).

[7] Ortiz AR, Strauss CEM, Olmea O (2002). MAMMOTH (matching molecular models obtained from theory): an automated method for model comparison. Protein Sci.

[8] Shindyalov IN, Bourne PE (1998). Protein structure alignment by incremental combinatorial extension (CE) of the optimal path. Protein Eng.

[9] Konagurthu AS, Whisstock JC, Stuckey PJ, Lesk AM (2006). MUSTANG: a multiple structural alignment algorithm. Proteins: Struct, Funct, Bioinf.

[10] Ye Y, Godzik A (2003). Flexible structure alignment by chaining aligned fragment pairs allowing twists. Bioinformatics.

[11] Zhang Y, Skolnick J (2005). TM-align: a protein structure alignment algorithm based on the TM-score. Nucleic Acids Res.

[12] Gibrat J-F, Madej T, Bryant SH (1996). Surprising similarities in structure comparison. Curr Opin Struct Biol.

[13] Orengo CA, Taylor WR. SSAP: sequential structure alignment program for protein structure comparison. *Computer methods for macromolecular sequence analysis*. 1996. 10.1016/s0076-6879(96)66038-8 8743709

[14] Guerler A, Knapp EW (2008). Novel protein folds and their nonsequential structural analogs. Protein Sci.

[15] Stivala A, Wirth A, Stuckey PJ (2009). Tableau-based protein substructure search using quadratic programming. BMC bioinformatics.

[16] Schwede T, Peitsch MC (2008). Computational structural biology: Methods and applications.

[17] Wiederstein M, Gruber M, Frank K, Melo F, Sippl MJ (2014). Structure-based characterization of multiprotein complexes. Structure.

[18] Brohawn SG, Leksa NC, Spear ED, Rajashankar KR, Schwartz TU (2008). Structural evidence for common ancestry of the nuclear pore complex and vesicle coats. Science.

[19] Lesk AM (1995). Systematic representation of protein folding patterns. J Mol Graph.

[20] Konagurthu AS, Stuckey PJ, Lesk AM (2008). Structural search and retrieval using a tableau representation of protein folding patterns. Bioinformatics (Oxford, England).

[21] Konagurthu AS, Lesk AM (2013). Structure description and identification using the tableau representation of protein folding patterns. Methods in molecular biology (Clifton, NJ).

[22] Kamat AP, Lesk AM (2007). Contact patterns between helices and strands of sheet define protein folding patterns. Proteins.

[23] Murzin AG, Brenner SE, Hubbard T, Chothia C (1995). SCOP: a structural classification of proteins database for the investigation of sequences and structures. J Mol Biol.

[24] Chen K, Ruan J, Kurgan L (2006). Prediction of three dimensional structure of calmodulin. Protein J.

[25] Shatsky M, Nussinov R, Wolfson HJ (2002). Flexible protein alignment and hinge detection. Proteins: Struct, Funct, Bioinf.

[26] Devos D, Dokudovskaya S, Alber F, Williams R, Chait BT, Sali A (2004). Components of coated vesicles and nuclear pore complexes share a common molecular architecture. PLoS Biol.

[27] Field MC, Sali A, Rout MP (2011). Evolution: On a bender--BARs, ESCRTs, COPs, and finally getting your coat. J Cell Biol.

[28] Frishman D, Argos P (1995). Knowledge‐based protein secondary structure assignment. Proteins: Struct, Funct, Bioinf.

[29] Mizuguchi K, Deane CM, Blundell TL, Overington JP (1998). HOMSTRAD: a database of protein structure alignments for homologous families. Protein Sci.

[30] Slater AW, Castellanos JI, Sippl MJ, Melo F (2013). Towards the development of standardized methods for comparison, ranking and evaluation of structure alignments. Bioinformatics (Oxford, England).

[31] Fox NK, Brenner SE, Chandonia JM (2014). SCOPe: Structural Classification of Proteins--extended, integrating SCOP and ASTRAL data and classification of new structures. Nucleic Acids Res.

[32] Kabsch W, Sander C (1983). Dictionary of protein secondary structure: pattern recognition of hydrogen-bonded and geometrical features. Biopolymers.

[33] Jung J, Lee B (2000). Protein structure alignment using environmental profiles. Protein Eng.

[34] Carpentier M, Brouillet S, Pothier J (2005). YAKUSA: a fast structural database scanning method. Proteins.

[35] Kolodny R, Koehl P, Levitt M (2005). Comprehensive evaluation of protein structure alignment methods: scoring by geometric measures. J Mol Biol.

[36] Wall ME, Rechtsteiner A, Rocha LM. Singular value decomposition and principal component analysis. In: *A practical approach to microarray data analysis.* Springer. 2003: 91–109.

[37] Sung W-K. Algorithms in bioinformatics: A practical introduction: CRC Press; 2009. Broken Sound Parkway, NW Suite 300, Boca Raton, FL, 33487. USA.

[38] Smith TF, Waterman MS (1981). Identification of common molecular subsequences. J Mol Biol.

[39] Sippl MJ (2008). On distance and similarity in fold space. Bioinformatics (Oxford, England).

[40] Kabsch W (1978). A discussion of the solution for the best rotation to relate two sets of vectors. Acta Crystallogr A.

[41] Kearsley SK (1989). On the orthogonal transformation used for structural comparisons. Acta Crystallogr A.

[42] Wolda H (1981). Similarity indices, sample size and diversity. Oecologia.

[43] Fawcett T (2004). ROC graphs: Notes and practical considerations for researchers. Mach Learn.

[44] Vergara IA, Norambuena T, Ferrada E, Slater AW, Melo F (2008). StAR: a simple tool for the statistical comparison of ROC curves. BMC bioinformatics.

