# Supplementary material for: Efficient and automated large-scale detection of structural relationships in proteins with a flexible aligner
Source: BMC Bioinformatics. 2016 Jan 5;17:20. doi: 10.1186/s12859-015-0866-8 (PMC4702403; doi:10.1186/s12859-015-0866-8)
Supplement: Additional file 1: — Suplementary data, including: Table S1. Average QS values for best combinations of MOMA parameter values; Table S2. Comparison of structural alignments generated by MOMA with those defined in HOMSTRAD; Table S3. Benchmark test to assess the performance of MOMA; Table S4. Statistical analysis for the benchmark of MOMA with other methods; Table S5. Statistical analysis for the benchmark of MOMA with different methods to assign secondary structure; Table S6. Set of 100 distant homologous protein pairs obtained from HOMSTRAD database; Figure S1. Calibration of distance cutoff using the HOMSTRAD set with the best combination of parameter values; Figure S2. ROC curves for the small set of seven most common folds according to TOPS database; Figure S3. ROC curves of classification at the SCOP fold and superfamily level; Figure S4. Execution time of MOMA; Figure S5. Algorithm used for extracting the rigid local matches. (PDF 2793 kb) [file 12859_2015_866_MOESM1_ESM.pdf]

Supplementary Table S1. Average QS values for best combinations of MOMA parameter values.

g1	g2	D	Average QS
-4	-4	20	0.9436
-4	-3	20	0.9433
-3	-3	20	0.9432
-4	-5	20	0.9428
-6	-6	24	0.9423
-2	-3	20	0.9422
-2	-5	20	0.9422
-5	-11	28	0.9422
-6	-9	28	0.9422
-2	-4	20	0.9420
-2	-6	20	0.9420
-2	-8	20	0.9420
-3	-5	28	0.9420
-5	-10	28	0.9420
-6	-8	28	0.9420
-6	-7	24	0.9417
-6	-8	24	0.9417
-4	-1	20	0.9416
-5	-6	24	0.9416
-5	-12	28	0.9415
-5	-13	28	0.9415
-5	-14	28	0.9415
-6	-10	28	0.9415
-3	-1	20	0.9414
-6	-8	30	0.9414
-4	-8	30	0.9414
-6	-4	28	0.9413
-6	-5	28	0.9413
-6	-6	28	0.9413
-3	-7	20	0.9412

Only the 30 best average QS values are shown out of 4,000 combinations of parameter values tested. g1, g2: gap-opening penalties for the first and second step of the dynamic programming algorithm, respectively. These parameter values were varied in the range [-1, -20] with a step of -1. C: constant used to modulate the angular difference between a pair of secondary structure elements (SSEs) in the scoring scheme of the dynamic programming algorithm. This parameter value was 45. D: maximum distance threshold to define a contact between a pair of SSEs. This parameter value was varied in the range [12, 30] with a step of 2.

Supplementary Table S2. Comparison of structural alignments generated by MOMA with those defined in HOMSTRAD.

Query	Target	SSE pairs reported by MOMA	SSE pairs reported by HOMSTRAD	SSE pairs in common	QS index
154l	1qsaa	5	5	5	1.000
1a0i	1fvia	12	12	11	0.917
1a41	1a31a	12	11	11	0.957
1a48	1kuta	15	15	14	0.933
1a7j	1esma	13	13	13	1.000
1a9na	1d0ba	8	8	7	0.875
1ab4	1bjt	27	28	26	0.945
1al3	1i6aa	14	13	12	0.889
1am2	1at0	11	11	11	1.000
1amua	1lci	34	34	34	1.000
1b3qa	1bxda	8	8	8	1.000
1b63a	1h7sa	18	19	18	0.973
1b74a	1jfla	14	15	12	0.828
1b8ba	3pfla	22	23	21	0.933
1b9ia	1jg8a	20	20	20	1.000
1bf2	1ehaa	22	22	21	0.955
1bgla	1bhga1	14	14	14	1.000
1bgyb	1bgya	21	22	21	0.977
1bhga2	1dp0a	10	8	8	0.889
1bk0	1dcs	19	19	19	1.000
1bp12	1bp11	11	9	7	0.700
1bs9	1cex	11	11	10	0.909
1by5a	1fepa	29	29	29	1.000
1clc	1tf4b	16	16	16	1.000
1cqea	1mhla	20	19	19	0.974
1ct5a	1bd0a	16	16	16	1.000
1d2ra	2ts1	15	17	12	0.750
1dhpa	1nal1	20	20	20	1.000
1dpe	1jeta	30	30	28	0.933
1dqaa	1qaya	23	24	22	0.936
1dqwa	1dbta	15	15	15	1.000
1e42a	1qtsa	15	15	15	1.000
1eg2a	1boo	14	15	14	0.966
1egua	1cb8a	39	38	37	0.961
1ejea	1i0ra	9	11	9	0.900
1em2a	1jssa	12	12	12	1.000
1emsa	1fo6a	20	20	20	1.000
1erja	1gotb	29	29	28	0.966
1evsa	1lki	5	4	4	0.889
1ezia	1h7ea	13	13	13	1.000
1f8sa	1h83a	30	30	26	0.867
1fi4a	1h72c	20	18	17	0.895
1fioa	1ez3a	3	3	3	1.000
1fm2a	1e3aa	37	37	34	0.919
1fmta	2gar	11	11	11	1.000
1fo4a	1ffvc	19	19	19	1.000
1fs0g	1e79g	10	10	10	1.000
1ft1a	1dcea	15	15	15	1.000
1fxxa	1j54a	12	12	12	1.000
1g6ga	1qu5a	8	6	2	0.286
1g6sa	1ejda	33	32	32	0.985
1ga8a	1ll3a	18	18	15	0.833

Query	Target	SSE pairs reported by MOMA	SSE pairs reported by HOMSTRAD	SSE pairs in common	QS index
1gcua	1ofga	21	21	21	1.000
1ggxa	1gfla	12	12	12	1.000
1gln	1qtqa	18	17	16	0.914
1gr0a	1jkia	19	19	18	0.947
1gvfa	1dosa	19	19	19	1.000
1hu3a	1h6ka	10	9	9	0.947
1i7da	1cy9a	12	12	12	1.000
1igra2	1igra1	5	6	5	0.909
1iira	1f0ka	24	24	24	1.000
1iq0a	1f7ua	29	31	29	0.967
1iq8a	1k4ga	21	22	21	0.977
1jcua	1hrua	13	13	13	1.000
1jdia	1fua	13	13	13	1.000
1jeyb	1jeya	25	25	25	1.000
1jgta	1ct9a	31	31	31	1.000
1jj2e	1rl6a	14	14	14	1.000
1jj2j	1whi	6	6	6	1.000
1jmkc	1keza	11	11	11	1.000
1jqra	1bpya	13	13	13	1.000
1js8a	1bt3a	11	10	10	0.952
1k6da	1poia	16	16	16	1.000
1kas	1afwa	19	17	17	0.944
1kit2	1kit1	12	12	12	1.000
1l5ja	1c96a	26	26	26	1.000
1l8aa	1trka	16	16	16	1.000
1lam	1gyta	10	10	10	1.000
1lnsa	1ju3a	31	31	31	1.000
1m2vb	1m2oa	35	36	35	0.986
1moq2	1moq1	10	11	10	0.952
1n1ma	1h2wa	17	16	15	0.909
1n2za	1efdn	16	15	14	0.903
1pbe	1foha	23	23	23	1.000
1pbwa	1tx4a	10	10	10	1.000
1qgia	1chka	11	11	8	0.727
1qhta	1ih7a	20	20	19	0.950
1rdr	1khva	18	17	17	0.971
1rkd	1bx4a	22	22	22	1.000
1rmg	1bhe	22	22	11	0.500
1thfd	1qo2a	17	17	17	1.000
1uag	1fgs	14	15	13	0.897
1vsga	2vsga	10	10	7	0.700
1yaca	1nbaa	12	12	12	1.000
2abk	1mun	10	10	9	0.900
2pgi	1dqra	25	27	24	0.923
2sqca	1ft1b	13	12	12	0.960
2tmda	1oyc	19	19	19	1.000
2tysb	1tdj	21	21	20	0.952

MOMA alignments were calculated with the best combination of parameter values (g1 = -4, g2 = -4, C = 45, D = 20). The alignments highlighted in red color represent those cases where MOMA generated a bad superposition (QS index <= 0.5).

Supplementary Table S3. Benchmark test to assess the performance of MOMA.

SCOP id	Fold	SCOP levels	# SSE	AUC						Execution Time (s)
				Raw score			Relative similarity			
				Fold	Superfamily	Family	Fold	Superfamily	Family	
d1ubia_	β-grasp	d.15.1.1	6	0.96	0.98	0.98	0.99	0.99	0.99	3.14
d1tttb1	Key-barrel	b.43.3.1	8	0.95	0.93	0.96	0.96	0.95	0.98	3.82
d1ae6h1	Immunoglobulin	b.1.1.1	10	0.94	0.96	0.98	0.97	0.98	0.98	4.59
d1bhne	Plait	d.58.6.1	12	0.92	1.00	1.00	0.97	1.00	1.00	6.01
d1h6rb_	GFP-like	d.22.1.1	14	1.00	1.00	1.00	1.00	1.00	1.00	7.20
d1tima_	Tim-barrel	c.1.1.1	20	0.99	1.00	0.99	0.99	1.00	1.00	12.40
d1f6dc_	NAD-binding fold	c.87.1.3	28	0.99	0.99	1.00	0.99	0.99	1.00	21.46

Area under ROC curves and execution times of MOMA when using the small set of seven most common folds as queries against the 19,602 domains in ASTRAL 95% sequence identity dataset. Calculations were carried out on a computer with an Intel Core i7 2.64 GHz processor with 12 GB memory, running Ubuntu 13.04 Linux system. The total execution time is reported in seconds.

Supplementary Table S4. Statistical analysis for the benchmark of MOMA with other methods (fold and superfamily levels). This analysis was realized with StAR server (http://melolab.org/star/home.php). In the diagonal AUC values are shown (bold type). The values reported in the upper-right triangle correspond to AUC differences of each pairwise comparison of methods. In the lower-left triangle are P-values of Mann-Whitney U-statistic non-parametric test for the AUC differences calculated. The values in green indicate that the observed differences are statistical significant (α=0.05), otherwise the values are shown in red.

Fold level

Methods	Structal	TopMatch	MOMA	GANGSTA+	QPtableau	SHEBA	Yakusa	FATCAT flex
**Structal**	***0.9557***	0.00019	0.01557	0.03900	0.07890	0.08551	0.16544	0.11838
**TopMatch**	0.93415	***0.9555***	0.01538	0.03882	0.07871	0.08532	0.16525	0.11819
**MOMA**	< 1E-05	0.00021	***0.9401***	0.02344	0.06333	0.06994	0.14987	0.10281
**GANGSTA+**	< 1E-05	< 1E-05	< 1E-05	***0.9167***	0.03990	0.04650	0.12643	0.07937
**QPtableau**	< 1E-05	< 1E-05	< 1E-05	< 1E-05	***0.8768***	0.00661	0.08654	0.03948
**SHEBA**	< 1E-05	< 1E-05	< 1E-05	< 1E-05	0.14672	***0.8702***	0.07993	0.03287
**YAKUSA**	< 1E-05	< 1E-05	< 1E-05	< 1E-05	< 1E-05	< 1E-05	***0.7902***	0.04706
**FATCATflex**	< 1E-05	< 1E-05	< 1E-05	< 1E-05	< 1E-05	< 1E-05	< 1E-05	***0.8373***

Superfamily level

Methods	Structal	TopMatch	MOMA	GANGSTA+	QPtableau	SHEBA	Yakusa	FATCAT flex
**Structal**	***0.9696***	0.00409	0.01319	0.05801	0.05197	0.08021	0.11199	0.05817
**TopMatch**	0.07733	***0.9737***	0.01729	0.06211	0.05606	0.08431	0.11608	0.06227
**MOMA**	< 1E-05	0.00009	***0.9564***	0.04482	0.03877	0.06702	0.09879	0.04498
**GANGSTA+**	< 1E-05	< 1E-05	< 1E-05	***0.9116***	0.00604	0.02220	0.05398	0.00016
**QPtableau**	< 1E-05	< 1E-05	< 1E-05	0.21668	***0.9176***	0.02825	0.06002	0.00621
**SHEBA**	< 1E-05	< 1E-05	< 1E-05	< 1E-05	< 1E-05	***0.8894***	0.03177	0.02204
**YAKUSA**	< 1E-05	< 1E-05	< 1E-05	< 1E-05	< 1E-05	< 1E-05	***0.8576***	0.05381
**FATCATflex**	< 1E-05	< 1E-05	< 1E-05	0.97525	0.18959	0.0018	< 1E-05	***0.9114***

Supplementary Table S5. Statistical analysis for the benchmark of MOMA with different methods to assign secondary structure (DSSP and Stride). Each set contains 10,000 pairs (5,000 positive and 5,000 negative cases) that were randomly selected from the original set (ASTRAL SCOP 2.3 40% sequence identity dataset). The statistical analysis was carried out with StAR server (http://melolab.org/star). The values in red indicate that the observed differences are not statistical significant (α=0.05).

**SCOP classification**	**Fold**		**Superfamily**		**Family**	
**Methods**	**DSSP**	**Stride**	**DSSP**	**Stride**	**DSSP**	**Stride**
**Area under ROC curve (AUC)**	0.9521	0.9521	0.9582	0.9567	0.9835	0.9833
**Accuracy**	0.8846	0.8844	0.8933	0.8911	0.9376	0.9390
**Optimal Threshold (Sr)**	22.7	22.3	23.4	25.8	30.9	30.3
**False positive rate**	0.1148	0.1266	0.1100	0.0884	0.0518	0.0594
**True positive rate**	0.8890	0.8954	0.8966	0.8706	0.9270	0.9374
**AUC differences**	0.0009		0.0015		0.0002	
**p-value**	0.4685		0.1800		0.8017	

Sr: percentage of relative similarity (Sippl 2008)

Supplementary Table S6. Set of 100 distant homologous protein pairs obtained from HOMSTRAD database.

Query	Target	Number of SSE	HOMSTRAD family
154l	1qsaa	5	Transglycosylase SLT domain
1a0i	1fvia	12	DNA ligase
1a41	1a31a	11	Topoisomerase I core
1a48	1kuta	15	SAICAR synthetase
1a7j	1esma	13	Phosphoribulokinase/Uridine kinase
1a9na	1d0ba	8	Leucine rich repeats in splicesomal and internalin B
1ab4	1bjt	28	Type II DNA topoisomerase
1al3	1i6aa	13	LysR
1am2	1at0	11	Hint (Hedgehog/Intein)
1amua	1lci	34	AMP binding
1b3qa	1bxda	8	Histidine kinase
1b63a	1h7sa	19	DNA mismatch repair protein
1b74a	1jfla	15	Asp/Glu/Hydontoin racemase
1b8ba	3pfla	23	Glycine radical
1b9ia	1jg8a	20	DegT/DnrJ/EryC1/StrS
1bf2	1ehaa	22	Isoamylase and glycosyltrehalose trehalohydrolase
1bgla	1bhga1	14	Glycosyl hydrolase family 2
1bgyb	1bgya	22	Insulinase
1bhga2	1dp0a	8	Glycosyl hydrolase family 2, sugar binding domain
1bk0	1dcs	19	Iron/Ascorbate oxidoreductase
1bp12	1bp11	9	LBP/BPI/CETP
1bs9	1cex	11	Cutinase
1by5a	1fepa	29	TonB-dependent receptor proteins
1clc	1tf4b	16	Glycosyl hydrolases family 9
1cqea	1mhla	19	Animal haem peroxidase
1ct5a	1bd0a	16	Alanine racemase, N-terminal domain
1d2ra	2ts1	17	Tyrosyl-tRNA synthetase
1dhpa	1nal1	20	Dihydrodipicolinate synthetase
1dpe	1jeta	30	Bacterial extracellular solute-binding proteins, family 5
1dqaa	1qaya	24	HMG-CoA reductase
1dqwa	1dbta	15	Orotidine 5'-phosphate decarboxylases
1e42a	1qtsa	15	Alpha adaptin AP2, C-terminal
1eg2a	1boo	15	DNA methylase
1egua	1cb8a	38	Polysaccharide lyase family 8
1ejea	1i0ra	11	Flavin reductase like domain
1em2a	1jssa	12	START domain
1emsa	1fo6a	20	Carbon-nitrogen hydrolase
1erja	1gotb	29	WD domain, G-beta repeat
1evsa	1lki	4	LIF/OSM
1ezia	1h7ea	13	Cytidylyltransferase
1f8sa	1h83a	30	Flavin containing amine oxidase
1fi4a	1h72c	18	GHMP kinases putative ATP-binding protein
1fioa	1ez3a	3	Syntaxin
1fm2a	1e3aa	37	Penicillin amidase
1fmta	2gar	11	Formyl transferase
1fo4a	1ffvc	19	FAD binding domain in molybdopterin dehydrogenase
1fs0g	1e79g	10	ATP synthetase g
1ft1a	1dcea	15	Protein prenyltransferase alpha subunit repeat

Query	Target	Number of SSE	HOMSTRAD family
1fxxa	1j54a	12	Exonuclease
1g6ga	1qu5a	6	FHA domain
1g6sa	1ejda	32	EPSP synthase
1ga8a	1ll3a	18	Glycosyl transferase family 8
1ggxa	1gfla	12	Green/red fluorescent protein
1gln	1qtqa	17	tRNAsynthetase 1c
1gr0a	1jkia	19	Inositol 1-phosphate synthetase
1gtta	1hyoa	11	FAA hydrolase
1gvfa	1dosa	19	Fructose-bisphosphate aldolase class II
1hu3a	1h6ka	9	MIF4G domain
1i7da	1cy9a	12	DNA topoisomerase
1igra2	1igra1	6	Receptor L domain
1iira	1f0ka	24	Glycosyltransferase family 28
1iq0a	1f7ua	31	tRNAsynthetase 1d
1iq8a	1k4ga	22	Queuine tRNA-ribosyltransferase
1jcua	1hrua	13	yrdC domain
1jdia	1fua	13	Class II Aldolase
1jeyb	1jeya	25	KU domain
1jgta	1ct9a	31	Asparagine synthase
1jj2e	1rl6a	14	Ribosomal protein L6
1jj2j	1whi	6	Ribosomal protein L14
1jmkc	1keza	11	Thioesterase domain
1jqra	1bpya	13	DNA polymerase X
1js8a	1bt3a	10	Tyrosinase
1k6da	1poia	16	Coenzyme A transferase
1kas	1afwa	17	Thiolase
1kit2	1kit1	12	Sialidase N terminal
1l5ja	1c96a	26	Aconitase
1l8aa	1trka	16	Transketolase
1lam	1gyta	10	Cytosol aminopeptidase
1lnsa	1ju3a	31	Peptidase S15
1m2vb	1m2oa	36	Sec23/Sec24
1moq2	1moq1	11	Isomerase domain
1n1ma	1h2wa	16	Peptidase S9
1n2za	1efdn	15	Periplasmic binding protein
1pbe	1foha	23	PHBH-like
1pbwa	1tx4a	10	GTPase-activator protein for Rho-like GTPases
1qgia	1chka	11	Glycosyl hydrolase family 46
1qhta	1ih7a	20	DNA polymerase family B, C-terminal
1rdr	1khva	17	RNA dependent RNA polymerase
1rkd	1bx4a	22	Ribokinase-like
1rmg	1bhe	22	Glycosyl hydrolase family 28
1thfd	1qo2a	17	Histidine biosynthesis protein
1uag	1fgs	15	Mur ligase
1vsga	2vsga	10	Trypanosome variant surface glycoprotein
1yaca	1nbaa	12	Isochorismatase
2abk	1mun	10	Endonuclease III
2pgi	1dqra	27	Phosphoglucose isomerase
2sqca	1ft1b	12	Prenyltransferase and squalene oxidase repeats
2tmda	1oyc	19	FMN oxidoreductase
2tysb	1tdj	21	Pyridoxal-phosphate dependent enzymes

These alignments were used to calibrate some parameter values of MOMA. The total number of matching secondary structural element (SSE) pairs, according to HOMSTRAD structural superpositions and STOVCA structural alignment derivations with default parameter values, are shown. In the last column, the family name of each protein pair is displayed.

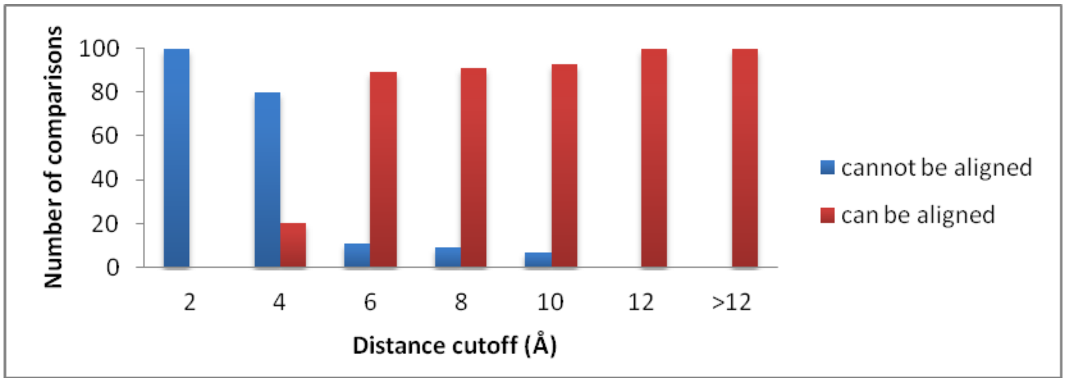

Supplementary Figure S1. Calibration of distance cutoff using the HOMSTRAD set with the best combination of parameter values. Histogram shows the number of comparisons carried out with MOMA varying the distance cutoff to define a contact between two secondary structure elements in the matrices. Blue and red bars indicate the comparisons that cannot and can be aligned using MOMA, respectively. The comparisons were carried out with the best combination of parameter values.

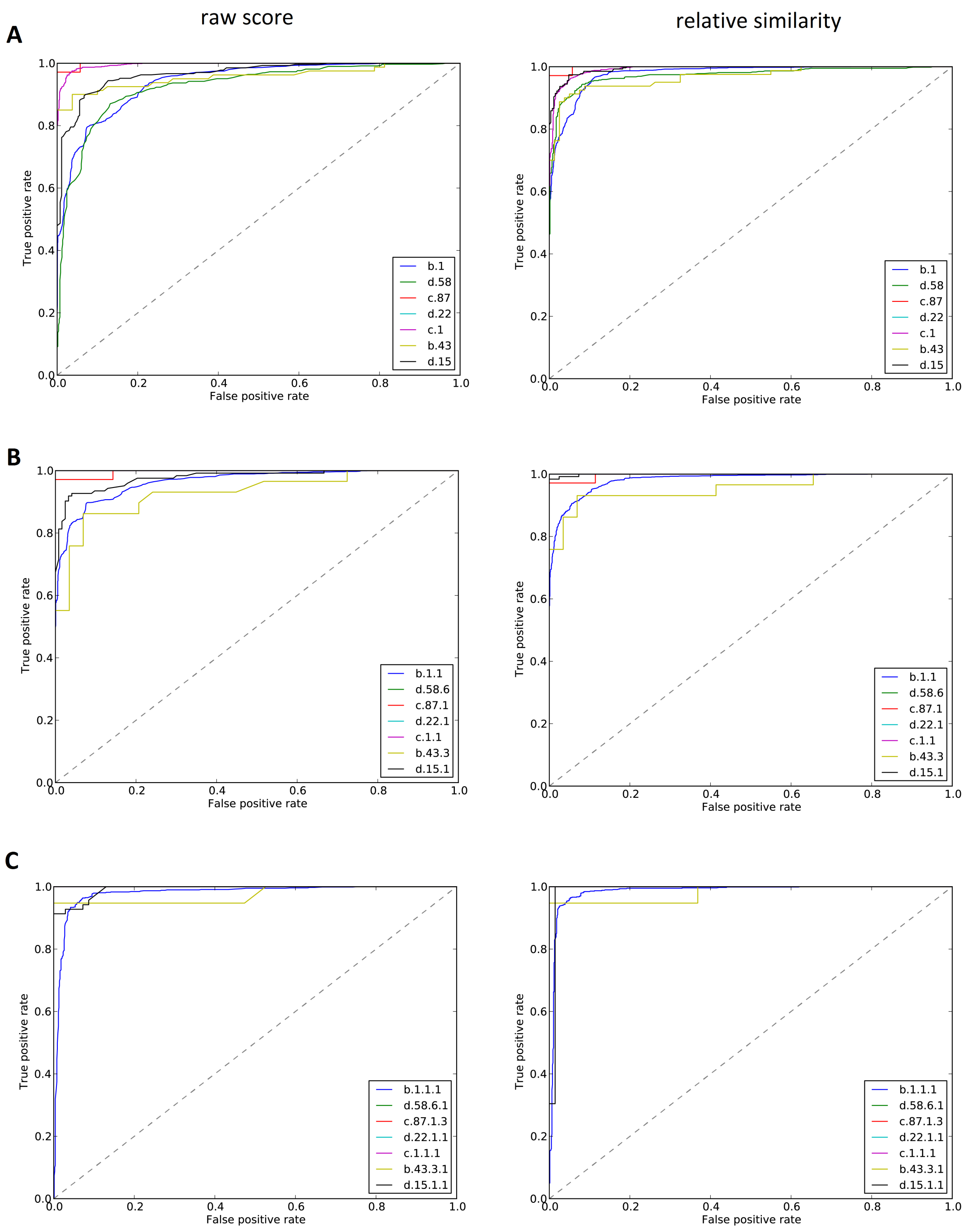

Supplementary Figure S2. ROC curves for the small set of seven most common folds according to TOPS database using raw score S (left) and relative similarity S_r (right) from MOMA. The searches were realized against ASTRAL 2.3 95% identity sequence, using the fold (A), superfamily (B) and family (C) levels as defined in SCOP database. The naming scheme of these proteins is from SCOP.

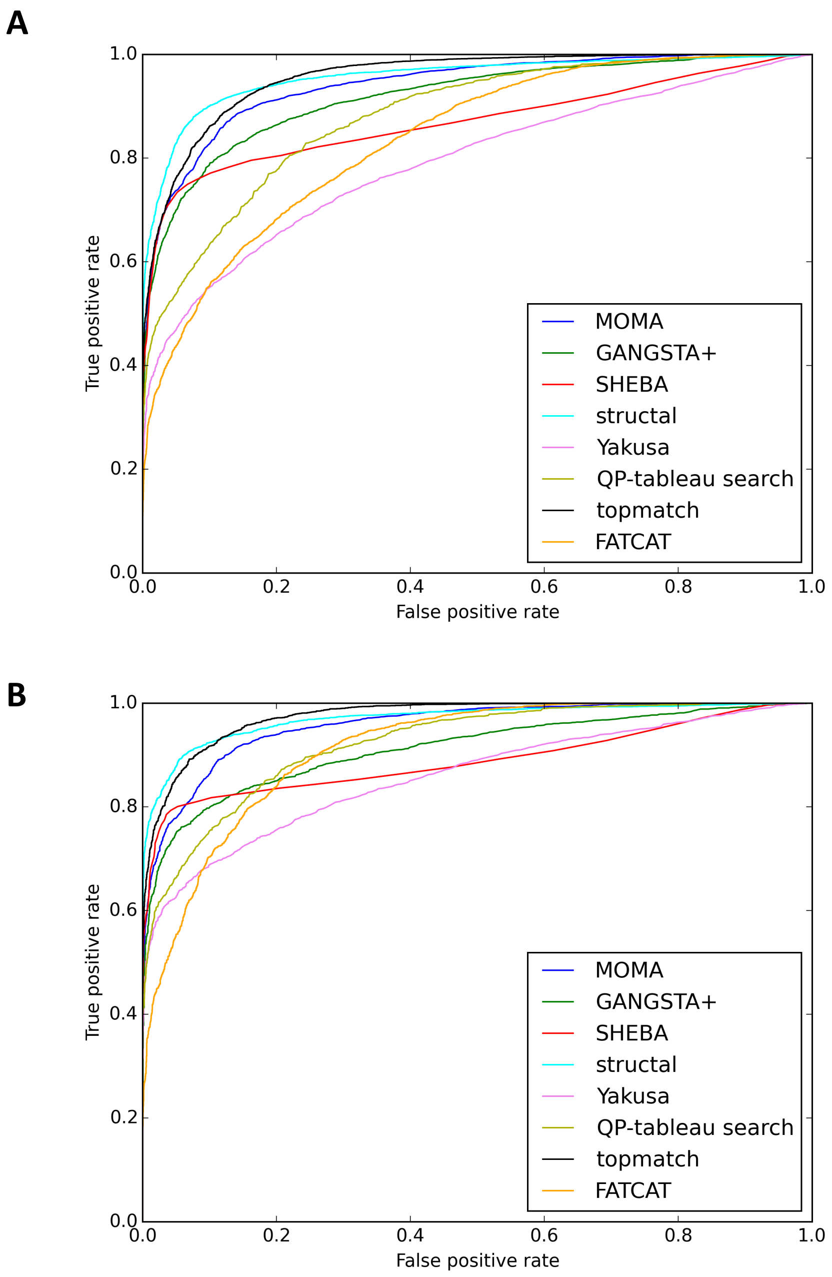

Supplementary Figure S3. ROC curves of classification at the SCOP fold (A) and superfamily level (B) for the large set of 100 proteins compared with other methods against ASTRAL SCOP 2.3 40% sequence identity dataset.

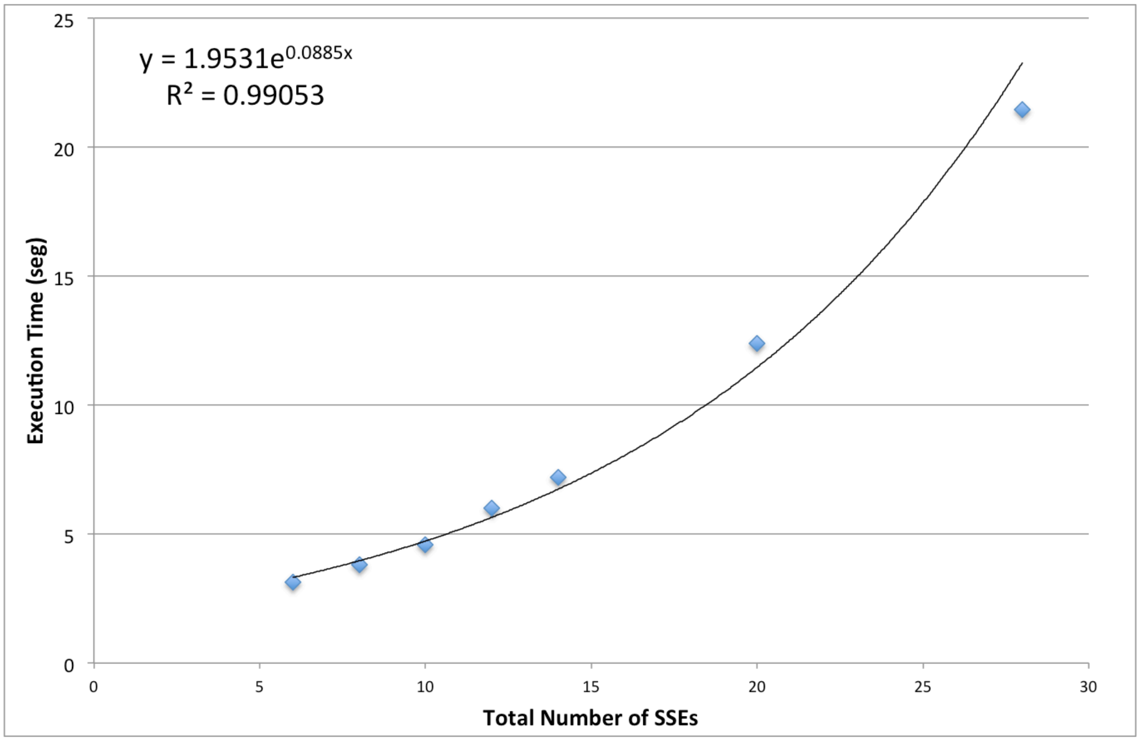

Supplementary Figure S4. Execution time of MOMA when varying the number of SSE considered, using the seven most common folds as a query. The searches were realized against ASTRAL 2.3 95% identity sequence, using the fold, superfamily and family levels as defined in SCOP database.

Algorithm: Method for generating blocks
Inputs: ΔSM, the Δ submatrix; N, the number of rows (Δ submatrix is a square matrix)
Output: B, a list of blocks
Require: N > 0, C > 0

```
C ← 90
B ← [ ]
end_block ← 1
nb ← 0                          # Size of block
i ← 0
while i < N do
        j ← 0
        if i = 0 then
        pi ← i                  # Position i of block
        pj ← i                  # Position j of block
        end if
        while j < i do
                if j = i - 1 then
                        if ΔSM i,j ≥ C and ΔSM i,j = NULL then
                                end_block ← 1
                                k ← j
                                while k ≥ 0 do
                                        if ΔSM i,k ≠ NULL and ΔSM i,k < C then
                                                end_block ← 0
                                                break while
                                        end if
                                        k ← k - 1
                                end while
                                if end_block = 1 then
                                        k = i + 1
                                        while k < N do
                                                if ΔSM k,j ≠ NULL and ΔSM k,j < C then
                                                        end_block ← 0
                                                        break while
                                                end if
                                                k ← k + 1
                                        end while
                                end if
                        end if
                        nb ← nb + 1
                end if
                if end_block = 1 then
                        if nb > 2 then
                                push (pi, pj, nb) onto B
                        end if
                        nb ← 0
                        pi ← i
                        pj ← i
                end if
                j ← j + 1
                end while
        i ← i + 1
end while
if (nb + 1) > 2 then
        push (pi, pj, nb) onto B
end if
```

Supplementary Figure S5. Algorithm used for extracting the rigid local matches. Pseudocode of the algorithm used for the extraction of independent protein segments that will follow different geometrical transformations for the global flexible superposition of secondary structure elements. The algorithm extracts one or more contiguous rectangular blocks of SSE pairs.
